# The Female Martyr

**Published:** 1888-10-27

**Authors:** John G. Whittier


### THE FEMALE MARTYR.

[*Sister Mary, aged* 18, *died in one of the Atlantic cities during an epidemic of cholera, while in voluntary attendance on the sick. Wrapt in a sheet, she was cast into a common grave.*]

For thou wast one in whom the light
Of Heaven's own love was kindled well,
Enduring with a martyr's might,
Through weary day and wakeful night,
Far more than words can tell;
Gentle and meek, and lowly and unknown,
Thy mercies measured by thy God alone!

Where manly hearts were failing, where
The throngful street grew foul with death,
Oh, high souled martyr! thou wast there
Inhaling from the loathsome air
Poison with every breath;
Yet shrinking not from offices of dread
From the wrung dying and the unconscious dead.

Sleep on in peace. The earth has not
A nobler name than thine shall be.
The deeds by martial manhood wrought,
The lofty energies of thought,
The fire of poesy—
These have but frail and fading honours; thine
Shall time unto eternity consign.

John G. Whittier.

